# Digital PET compliance to EARL accreditation specifications

**DOI:** 10.1186/s40658-017-0176-5

**Published:** 2017-01-31

**Authors:** Daniëlle Koopman, Maureen Groot Koerkamp, Pieter L. Jager, Hester Arkies, Siert Knollema, Cornelis H. Slump, Pedro G. Sanches, Jorn A. van Dalen

**Affiliations:** 1 Department of Nuclear Medicine, Isala Hospital, Zwolle, the Netherlands; 2 MIRA Institute for Biomedical Technology and Technical Medicine, University of Twente, Enschede, the Netherlands; 3 Health Systems, Philips Benelux, Eindhoven, the Netherlands; 4 Department of Medical Physics, Isala Hospital, Zwolle, the Netherlands

**Keywords:** Digital PET, EANM guidelines, EARL accreditation, FDG-PET, Tumour imaging

## Abstract

**Background:**

Our aim was to evaluate if a recently introduced TOF PET system with digital photon counting technology (Philips Healthcare), potentially providing an improved image quality over analogue systems, can fulfil EANM research Ltd (EARL) accreditation specifications for tumour imaging with FDG-PET/CT.

**Findings:**

We have performed a phantom study on a digital TOF PET system using a NEMA NU2-2001 image quality phantom with six fillable spheres. Phantom preparation and PET/CT acquisition were performed according to the European Association of Nuclear Medicine (EANM) guidelines. We made list-mode ordered-subsets expectation maximization (OSEM) TOF PET reconstructions, with default settings, three voxel sizes (4 × 4 × 4 mm^3^, 2 × 2 × 2 mm^3^ and 1 × 1 × 1 mm^3^) and with/without point spread function (PSF) modelling.

On each PET dataset, mean and maximum activity concentration recovery coefficients (RC_mean_ and RC_max_) were calculated for all phantom spheres and compared to EARL accreditation specifications. The RCs of the 4 × 4 × 4 mm^3^ voxel dataset without PSF modelling proved closest to EARL specifications. Next, we added a Gaussian post-smoothing filter with varying kernel widths of 1–7 mm. EARL specifications were fulfilled when using kernel widths of 2 to 4 mm.

**Conclusions:**

TOF PET using digital photon counting technology fulfils EARL accreditation specifications for FDG-PET/CT tumour imaging when using an OSEM reconstruction with 4 × 4 × 4 mm^3^ voxels, no PSF modelling and including a Gaussian post-smoothing filter of 2 to 4 mm.

## Introduction

Recently, a time-of-flight (TOF) positron emission tomography (PET) system was introduced by Philips Healthcare, with digital photon counting technology using silicon photomultipliers. The replacement of conventional photomultipliers by digital detectors, including the implementation of single-photon avalanche photodiodes, provides true digital photon counting without the need of additional analogue-to-digital conversions [1–3]. Moreover, the detector elements and the scintillator crystals have equal sizes which enables one-to-one coupling. Acceptance tests on performance characteristics showed that this digital PET provides a higher timing resolution and improved spatial resolution, as compared to state-of-the-art analogue PET using conventional photomultipliers [4, 5]. In clinical practice, digital PET may provide a higher image quality and improved small lesion detection and quantification [6].

PET/computed tomography (CT) scanning, using fluor-18 fluordeoxyglucose (FDG), has an important role in tumour imaging for patients with cancer. There is a trend towards standardization in FDG-PET scanning to allow quantitative comparisons of FDG-uptake parameters across patients, scanners and medical centres [7]. To support standardization between scanners and medical centres, the European Association of Nuclear Medicine (EANM) has published guidelines on FDG-PET tumour imaging [7, 8]. Furthermore, the EANM launched the EANM research Ltd (EARL) to promote nuclear medicine research and multi-centre studies. EARL has developed an accreditation programme for tumour imaging with FDG-PET/CT [9].

In clinical practice, the EARL FDG-PET/CT accreditation specifications are widely implemented. These specifications, which are primarily about activity concentration recovery coefficient (RC) measurements on PET images, are based on analogue PET systems using conventional photomultipliers [7, 8]. Intrinsically, higher RCs may be expected using digital PET, due to improved spatial and time-of-flight resolution compared to other non-digital, but state-of-the-art systems [5]. Our aim was to evaluate if a recently introduced TOF PET/CT system with digital photon counting technology can fulfil EARL requirements as well.

## Method

### Phantom study

We have performed a phantom study using a NEMA IEC-61675-1 NU2-2001 image quality phantom (IQ phantom) with six fillable spheres (10, 13, 17, 22, 28 and 37 mm diameter). According to the EANM guidelines [7], the IQ phantom was filled with FDG-activity, with a sphere-to-background ratio of 10:1. Using a TOF PET/CT system with digital photon counting technology (Philips Healthcare) [6], we performed a PET scan of one bed position with a scan duration of 10 min. Additionally, a CT scan was acquired for attenuation correction. Prior to our measurements, the digital PET was calibrated with FDG and verified to be within an offset of 2%, using the method as described in [7].

### PET reconstructions

We have performed six default TOF PET reconstructions, using blob-based ordered-subsets expectation maximization (OSEM) [10], with three voxel sizes and with/without point spread function (PSF) modelling, which corrects for partial-volume effects in PET images. When incorporating PSF modelling, we used a noise regularization kernel of 6 mm full-width at half-maximum and 1 PSF iteration. For each voxel size, we used a fixed number of iterations and subsets, as recommended by the manufacturer.

1. 4 × 4 × 4 mm^3^, with 3 iterations and 15 subsets, without PSF
2. 4 × 4 × 4 mm^3^, with 3 iterations and 15 subsets, with PSF
3. 2 × 2 × 2 mm^3^, with 3 iterations and 17 subsets, without PSF
4. 2 × 2 × 2 mm^3^, with 3 iterations and 17 subsets, with PSF
5. 1 × 1 × 1 mm^3^, with 3 iterations and 9 subsets, without PSF
6. 1 × 1 × 1 mm^3^, with 3 iterations and 9 subsets, with PSF

On each reconstructed PET dataset, we calculated mean and maximum activity concentration recovery coefficients (RC_mean_ and RC_max_) for all phantom spheres, according to EANM guidelines [7]. Next, we compared our RC results with EARL accreditation specifications [9].

To evaluate reconstruction settings for digital PET that meet the requirements for EARL accreditation, we performed additional reconstructions. We selected the reconstructed PET dataset whose RCs fitted best to the EARL accreditation specifications and added a 3D Gaussian post-smoothing filter with varying kernel widths of 1–7 mm, using standard vendor software. Again, RC_mean_ and RC_max_ were measured for all phantom spheres and compared to EARL accreditation specifications.

## Results

**Fig. 1:**
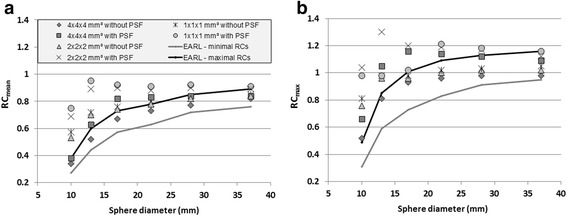
RC_mean_ and RC_max_ values for all phantom spheres, as compared to EARL minimal and maximal accreditation specifications. **a** RC_mean_ for six default TOF PET reconstructions. Only for a PET reconstruction using 4 × 4 × 4 mm^3^ voxels without PSF modelling, RC_mean_ values were all within accreditation specifications. **b** RC_max_ for six default TOF PET reconstructions. For all reconstructions, at least one RC_max_ was above maximal accreditation specifications

Figure 1 shows RC_mean_ and RC_max_ results for all spheres, for each of the six default PET reconstructions. For each reconstruction, at least one RC_max_ value was above EARL accreditation specifications. The PET reconstruction without PSF modelling and using 4 × 4 × 4 mm^3^ voxels showed RCs nearest to EARL requirements. For this specific reconstruction, only RC_max_ for the 10-mm sphere was above the EARL limit.

**Fig. 2:**
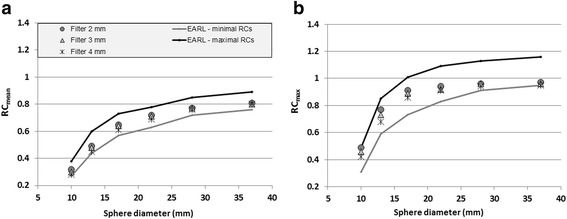
Impact of a Gaussian post-smoothing filter using a kernel width of 2, 3 and 4 mm on RC_mean_ (**a**) and RC_max_ (**b**) for a TOF PET reconstruction with 4 × 4 × 4 mm^3^ voxels without PSF modelling. For all phantom spheres, both RC_mean_ and RC_max_ fulfilled EARL accreditation specifications

Table 1 shows the impact of an additional Gaussian post-smoothing filter with 1 to 7 mm kernel widths, on RC_mean_ and RC_max_ in a PET reconstruction using 4 × 4 × 4 mm^3^ voxels, without PSF modelling. As shown in Fig. 2, EARL accreditation specifications for RC_mean_ and RC_max_ can be achieved with digital PET using filters with kernel widths of 2 to 4 mm.

**Table 1:** The impact of a Gaussian post-smoothing filter with a kernel width of 1–7 mm on RC_mean_ and RC_max_ for a TOF PET reconstruction with 4 × 4 × 4 mm^3^ voxels, without PSF modelling. RCs within EARL accreditation specifications are marked in italic style

Recon	EARL minimum	EARL maximum	No filter	Filter 1 mm	Filter 2 mm	Filter 3 mm	Filter 4 mm	Filter 5 mm	Filter 6 mm	Filter 7 mm
Sphere diameter (mm)										
	RC_mean_									
10	0.27	0.38	*0.34*	*0.33*	*0.32*	*0.30*	*0.28*	0.26	0.24	0.22
13	0.44	0.60	*0.52*	*0.51*	*0.49*	*0.48*	*0.45*	0.42	0.39	0.35
17	0.57	0.73	*0.67*	*0.65*	*0.65*	*0.64*	*0.61*	*0.58*	0.55	0.52
22	0.63	0.78	*0.73*	*0.72*	*0.72*	*0.71*	*0.69*	*0.67*	*0.64*	0.62
28	0.72	0.85	*0.77*	*0.77*	*0.77*	*0.77*	*0.76*	*0.74*	*0.72*	0.71
37	0.76	0.89	*0.82*	*0.81*	*0.81*	*0.80*	*0.80*	*0.78*	*0.77*	0.76
	RC_max_									
10	0.31	0.49	0.52	0.51	*0.49*	*0.46*	*0.42*	*0.38*	*0.34*	0.31
13	0.59	0.85	*0.81*	*0.80*	*0.77*	*0.73*	*0.68*	*0.63*	0.57	0.51
17	0.73	1.01	*0.93*	*0.92*	*0.91*	*0.89*	*0.86*	*0.82*	*0.77*	0.72
22	0.83	1.09	*0.96*	*0.96*	*0.94*	*0.92*	*0.91*	*0.90*	*0.89*	*0.87*
28	0.91	1.13	*0.98*	*0.98*	*0.96*	*0.96*	*0.94*	*0.93*	*0.92*	*0.91*
37	0.95	1.16	*0.98*	*0.98*	*0.97*	*0.96*	*0.95*	0.94	0.94	0.94

## Conclusion

PET with digital photon counting technology typically shows an activity concentration recovery coefficient above EARL specifications, especially for small objects. To meet EARL standards, a TOF OSEM reconstruction without PSF modulation, with 3 iterations, 15 subsets, 4 × 4 × 4 mm^3^ voxels and a Gaussian post-smoothing filter with a kernel width of 2 to 4 mm can be used.

## Discussion

To meet EARL standards for PET with digital photon counting technology, the use of relatively large 4 × 4 × 4 mm^3^ voxels and a post-smoothing filter is recommended. With smaller voxel sizes and/or PSF modelling, RCs in our study were above EARL specifications. This has been demonstrated for state-of-the-art analogue PET systems as well [11, 12]. With the introduction of advanced reconstruction algorithms (e.g. using small voxels or incorporating PSF modelling), eventually combined with new digital PET technologies, EARL specification updates may be needed in the future. Under the assumption that the availability and presence of PET scanners using older technology will decrease, a way to maintain the uniformity across modern PET cameras is to increase both lower- and upper RC EARL specifications, especially for small spheres. Furthermore, the use of smaller phantom spheres, for example as available in a micro phantom that we used in a previous study [11], may be warranted to be able to compare reconstruction algorithms for smaller sphere sizes and to harmonize the quantification of small lesions across scanners.

Besides, given the high RCs that can be achieved with digital photon counting technology combined with advanced reconstruction settings, it might be appropriate to perform multiple PET reconstructions for different purposes. Next to an EARL-approved reconstruction to perform quantitative analyses, a high-resolution small-voxel PET reconstruction could be made for visual evaluation and optimal small lesion detection [8, 11, 13].

This short communication focused on determing PET reconstruction settings to fulfil EARL RC specifications. However, to obtain the EARL accreditation, these reconstruction settings should be chosen to meet both RC requirements and specifications for the calibration QC [7], and EANM guidelines should be fully implemented in clinical practice [7, 8].
